# Clusters of carbohydrate-rich foods and associations with type 2 diabetes incidence: a prospective cohort study

**DOI:** 10.1186/s12937-023-00906-0

**Published:** 2023-12-18

**Authors:** Kjell Olsson, Esther González-Padilla, Suzanne Janzi, Anna Stubbendorff, Yan Borné, Stina Ramne, Ulrika Ericson, Emily Sonestedt

**Affiliations:** 1 Nutritional Epidemiology, Department of Clinical Sciences Malmö, Lund University, Jan Waldenströms gata 35, Malmö, SE-21428 Sweden; 2 Diabetes and Cardiovascular Disease-Genetic Epidemiology, Department of Clinical Sciences Malmö, Lund University, Jan Waldenströms gata 35, Malmö, SE-21428 Sweden

**Keywords:** K-means clustering, Epidemiology, Malmö Diet and Cancer Study, Type 2 Diabetes, Diet, Nutrition

## Abstract

**Background:**

About one in ten adults are living with diabetes worldwide. Intake of carbohydrates and carbohydrate-rich foods are often identified as modifiable risk factors for incident type 2 diabetes. However, strong correlation between food variables can make it difficult to identify true associations. The purpose of this study was to identify clusters of carbohydrate-rich foods and analyse their associations with type 2 diabetes incidence in the Malmö Diet and Cancer Study cohort in southern Sweden.

**Methods:**

Dietary intake of 26 622 participants was assessed using a validated three-part diet history method: a 7-day food diary, a 168-item food frequency questionnaire, and a 60-minute interview. K-means clustering analysis identified five clusters from 21 food variables. The Cox proportional hazard regression model was applied to calculate hazard ratios (HR) and 95% confidence intervals (CI) of the association between clusters and incident type 2 diabetes.

**Results:**

The cluster analysis resulted in five clusters; *high vegetables/low added sugar*, *high sugar-sweetened beverages*, *high juice*, *high fruit*, and *high refined carbohydrates/low fruit & vegetables* (reference). During mean follow-up of 18 years, 4046 type 2 diabetes cases were identified. After adjustment for potential confounding (including lifestyle, body mass index, and diet), a *high fruit* cluster (HR 0.86; 95% CI 0.78, 0.94) was inversely associated with type 2 diabetes compared to the reference cluster. No other significant associations were identified.

**Conclusions:**

A dietary pattern defined by a high intake of fruits was associated with a lower incidence of type 2 diabetes. The findings provide additional evidence of a potential protective effect from fruit intake in reducing type 2 diabetes risk. Future studies are needed to explore this association further.

**Supplementary Information:**

The online version contains supplementary material available at 10.1186/s12937-023-00906-0.

## Introduction

Globally, about 537 million adults were living with diabetes in 2021, and the prevalence is expected to reach 783 million by 2045 [1]. Type 2 diabetes is the most common type of diabetes, comprising over 90% of all cases. Diet constitutes a major modifiable risk factor for type 2 diabetes, affecting postprandial glucose levels, insulin resistance, body weight, and more [2]. Multiple studies have identified inverse associations between different dietary patterns and incidence of type 2 diabetes, including for the Mediterranean diet, low glycaemic diets, and various plant-based diets [3–6].

In the Malmo Diet and Cancer Study (MDCS), previous findings have concluded that a health conscious dietary pattern (high in fiber-rich bread, breakfast cereals, fruits, vegetables, fish, and low-fat yoghurt, and low in low-fiber bread) was associated with lower incidence of type 2 diabetes, while no significant associations were found for a dietary pattern in accordance with the Swedish nutrition recommendations [7, 8]. Furthermore, we have also identified associations between several carbohydrate-rich foods and type 2 diabetes risk, including a reduced risk from intake of fruits and an increased risk from sweets [9]. However, because of the moderate to strong correlation found between many of the dietary variables, the true associations with disease outcomes from individual foods or nutrients are difficult to differentiate. There is thus a need to study associations between patterns of carbohydrate intake and type 2 diabetes risk.

While previous studies have applied *a priori* approaches to study the carbohydrate quality of diets, such as the glycemic index or carbohydrate ratio indices, this is the first study, to our knowledge, to apply an *a posteriori* approach of cluster analysis to identify dietary patterns of carbohydrate-rich foods [10]. In contrast to the *a priori* approach, the *a posteriori* approach is not limited to the confirmation of known associations of diet and disease, and can thus be applied to explore and identify new dietary patterns and associations [11]. Few studies have applied cluster analysis to identify dietary patterns for the study of the association with type 2 diabetes risk, and an association has not always been identified [12–14]. Furthermore, these studies have often been limited to cross-sectional data, highlighting a need for more analyses of dietary patterns in prospective studies [15]. The aim of this study was to identify clusters of carbohydrate-rich food intake and analyse associations with incidence of type 2 diabetes in a large, prospective cohort in southern Sweden.

## Subjects and methods

### Population

Recruitment and baseline examinations of the Malmö Diet and Cancer Study were carried out between January 1991 and September 1996 in the municipality of Malmö in southern Sweden. A total of 30 446 participants were recruited from a source population of 74 138 individuals, encompassing all males born between 1923 and 1945, and all females born between 1923 and 1950, within the municipality. Recruitment was achieved through advertising and distribution of invitation letters. Individuals with limited Swedish proficiency or mental disability, hindering them from filling out the baseline questionnaire, were excluded from participation. Full description of the recruitment process has been published elsewhere [16]. For this specific study, we excluded participants who did not complete their dietary assessment (*n* = 2348), with prevalent diabetes at baseline (*n* = 1230), or with missing data on smoking habits, physical activity or level of education (*n* = 246). Excluded participants had a similar mean age as the study population, but a higher proportion of males and a higher mean BMI. The total population for this study was 26 622 participants (61% females) (Supplemental Fig. 1). The Malmö Diet and Cancer Study was granted ethical approval by the Regional Ethics Committee (LU 90 − 51). All participants provided their informed written consent.

### Dietary assessment

Dietary assessment was conducted during baseline examinations, using a validated, modified diet history method consisting of three parts [17–19]. (1) A one-week food diary, recording intakes of prepared meals (commonly lunch and dinner), cold beverages, and supplements. (2) A 168-item food frequency questionnaire (FFQ), assessing portion-sizes and consumption frequencies of foods eaten regularly during the previous year. (3) A 60-minute interview, further assessing portion-sizes, food choices, and methods of preparation. The three-part method was designed to assess all aspects of the participant’s diet while ensuring that no overlapping data were collected. In September 1994, due to slight changes in the coding of dietary data, the interview was shortened from 60 to 45 min. The modification did not have a major effect on the ranking of participants [20].

All the FFQs were checked for missing values, and each participant’s FFQ was compared with the food diary (according to pre-defined instructions) to confirm that there was no overlapping in the registered food consumption. Recipes for the meals recorded in the food diary were sourced from the computer software KOSTSVAR (AIVO AB, Solna, Sweden), and could be altered during the interview if needed.

The modified diet history method has previously shown good ranking validity compared with a reference method of 18-day weighed food records [17, 18]. The energy-adjusted Pearson correlation coefficients for males and females were 0.65 and 0.53 for vegetable intake, 0.60 and 0.77 for fruit, 0.74 and 0.73 for cereals, 0.50 and 0.58 for bread, and 0.69 and 0.51 for potatoes [18].

### Dietary data

Data on food intake from the food diary and the FFQ were registered as average intake in grams per day of individual foods. Energy and nutrient intakes were calculated using a food composition database with 1600 food items from the Swedish National Food Agency. Additional recipes and food items were added specifically for this cohort. Extreme and median values of total energy, nutrients and major food groups in the data, as well as extreme portion-sizes, were routinely checked for errors.

The 21 foods included in the cluster analysis were selected based on available data on foods with a high proportion of energy from carbohydrates and encompassed: boiled or baked potato, fried or deep-fried potato, fruits (including berries, canned fruits, and dried fruits), vegetables (including cooked vegetables), juice (from fruits and vegetables), sugar-sweetened beverages (all carbonated and non-carbonated beverages; except juices, dairy products and alcoholic beverages), pastries (all sweetened baked goods), chocolate, sweets (all sugar-sweetened candy, except chocolate), table sugar (all sugar products added to foods and drinks after preparation), ice cream, marmalade/honey/jam (including apple sauce), ketchup, flour, grains/cereals (< 10% fiber), grains/cereals (≥ 10% fiber), soft bread (< 4.5% fiber), soft bread (≥ 4.5% fiber), crisp bread (< 10% fiber), crisp bread (≥ 10% fiber), and rice/pasta. All values were expressed as intake in grams per 1000 kcal of energy intake.

Energy intake was expressed as kcal of total daily intake, while alcohol intake was expressed as percentage of total energy intake, and carbohydrate, protein, fat, monosaccharide, disaccharide, sucrose, and added sugar intakes were expressed as percentage of the non-alcohol energy intake. Added sugar was calculated by adding all intake of sucrose and monosaccharides, excluding sucrose and monosaccharides from fruits and vegetables (including juices) [21]. Intake of fiber (all types of dietary fiber), fish (excluding shellfish), red meat (including processed meat), and coffee were expressed as intake in grams per 1000 kcal of energy intake.

The EAT-Lancet index was recently developed and validated in our cohort and is based on a diet proposed by the EAT-Lancet Commission [22, 23]. The index is regarded as a healthy plant-based diet index with a range between 0 and 42 points. A higher index score indicates a greater adherence to the EAT-Lancet diet.

### Covariate data

During baseline examinations, blood pressure, weight, height, waist circumference and percentage of body fat (with a bioelectrical impedance method) were measured, and 45 ml blood samples were collected. Participants were asked to fill out a self-administered questionnaire, providing information on their level of education, occupation, physical activity, smoking habits, alcohol consumption, social network, current health, medical history, current medication, and disease in close relatives.

Physical activity was assessed as the self-reported time spent per week on 17 leisure-time activities. The duration of each activity was multiplied with their respective metabolic equivalent intensity factor. The individuals were subsequently divided into five categories based on their metabolic equivalent of task hours (MET-hours) per week [24]. Alcohol habits was divided by six levels of reported intake: non-consumption and quintiles of consumption. Smoking habits was divided into three categories: current smoker, former smoker and never smoker. Education was divided into six categories corresponding to the highest achieved level of education. Season was divided into four categories, corresponding to the season when baseline assessments were carried out: winter (January–March), spring (April–June), summer (July–September) and fall (October–December). Diet method version was introduced following a minor adjustment of the coding routines of dietary data in September 1994, and was divided into two categories: old and new. Misreporters were participants who potentially misreported their energy intake, defined as having a ratio of reported energy intake to basal metabolic rate outside the 95% confidence interval (CI) of their calculated physical activity level [25]. Diet changers were defined as participants who reported that they had altered their eating habits in the past (due to disease or other reason) [26].

Among participants who entered the study from November 1991 and February 1994, a random 50% sample (*n* = 12,445) was invited to also participate in the Malmö Diet and Cancer – Cardiovascular Cohort; a sub-cohort of the MDCS [27]. In participants who accepted the invitation (*n* = 5540), additional blood analyses were carried out, including HbA1c, plasma glucose, and insulin. Homoeostatic model assessment of insulin resistance (HOMA-IR) was determined by using the formula: fasting plasma insulin (mU/L) × blood glucose (mmol/L)/22.5.

### Endpoint data

Participants who developed diabetes during the follow-up period (until 31 December 2016) were identified through several different registers, as well as during rescreening of the cohort. A large proportion of cases were reported in more than one register. Both the Regional Diabetes 2000 Registry of Scania (15.2% of cases) and the Swedish National Diabetes Register (57.1% of cases) required a diabetes diagnosis by a physician, in accordance with the established diagnostic criteria (a fasting plasma glucose concentration of ≥ 7.0 mmol/L, or a fasting whole blood concentration of ≥ 6.1 mmol/L, on two different occasions) [28, 29]. Diabetes cases were also identified through the HbA1c Registry at Clinical Chemistry, Malmö (individuals with at least two HbA1c values ≥ 6.0%) (51.6% of cases), as well as through four national registries under the National Board of Health and Welfare: the Cause-of-Death Registry (8.8% of cases), the Swedish Hospital-based Outpatient Care (44.8% of cases), the Swedish National Inpatient Registry (ICD10 codes E10-E14 and O244-O249) (52.7% of cases), and the Swedish Prescribed Drug Registry (ATC code A10) (71.1% of cases) [30–32]. Additionally, diabetes cases were identified through the screening of sub-samples of participants in the Malmö Diet and Cancer Study cohort (1992–1994, 1997–2001 and 2007–2012) (2.8%, 7.8% and 11.1% of cases), as well as in the Malmö Preventive Project Cohort (2002–2006) (18.6% of cases). For this study, focusing on type 2 diabetes, we excluded cases who were registered as having developed type 1 diabetes, latent autoimmune diabetes in adults, secondary diabetes or other diabetes condition in the outcome variable. Participants registered with diabetes of unknown type were assumed to be of type 2 and were thus included.

### Statistical analysis

Statistical analyses were performed using IBM SPSS Statistics (version 26.0; IBM Corp., Armonk, NY, USA). The main analyses were performed for all participants (*n* = 26 622). The K-means clustering analysis included 21 energy-adjusted, carbohydrate-rich food variables. The analysis was repeated with two to eight clusters to determine the optimal number of clusters. Baseline characteristics of clusters were analyzed by applying the general linear model for continuous variables (adjusted for age, sex, diet method version, season, and energy intake, when applicable) and cross tabulation for categorical variables.

The Cox proportional hazard regression model was used to calculate hazard ratios (HR) of the association between type 2 diabetes incidence and clusters of dietary intake. The cluster with the most participants, and least defined by one specific food, was used as the reference in our main analysis. Subsequent analyses applying each of the other clusters as the reference were also conducted. Years of follow-up was used as the time scale. The covariates included in the models were chosen based on known associations with type 2 diabetes from literature and were assessed during baseline examinations. In the first model, adjustments were made for sex, age, diet method version, season, and total energy intake. The second model included further adjustment for physical activity, alcohol habits, smoking habits, and education, and the third model included additional adjustment for body mass index (BMI). The fourth model included additional adjustment for two dietary variables: coffee, and red meat. Intake of red meat and processed meat has been associated with an increased risk of type 2 diabetes, while coffee intake has been associated with a reduced risk [2, 33]. Interaction for sex was examined by adding an interaction term in the fourth model, combining the cluster variable with the sex variable, and analyzing each cluster separately against the reference cluster. Potential mediating effects of total carbohydrate intake, fiber intake, and added sugar intake were considered but did not meet the assumptions of mediation [34]. Sensitivity analyses were conducted where potential misreporters of energy intake, diet changers, and participants who developed diabetes within two years from enrolment were excluded from the fourth model in separate analyses.

## Results

### Cluster characteristics

The best *K* number of clusters in our analysis was deemed to be five, as this resulted in clearly distinct clusters with a good distribution of participants in each cluster (*n* > 1000) (Supplemental Fig. 2). Four of these five clusters were clearly defined by a higher consumption of one or two specific foods, and thus aptly labelled after their defining variables: *high vegetables/low added sugar*, *high sugar-sweetened beverages*, *high juice* and *high fruit* (Table 1). The fifth and largest cluster was labelled the *high refined carbohydrates/low fruit & vegetables* cluster and encompassed roughly half of all participants (51.2%).

**Table 1:** Baseline table by clusters of carbohydrate-rich foods in participants in the Malmö Diet and Cancer cohort (n = 26 622)1,2

Variables	Cluster membership				
	High refined carbs/Low fruit & veg	High vegetables/Low added sugar	High sugar-sweetened beverages	High juice	High fruit
	Mean (95% CI) or %	Mean (95% CI) or %	Mean (95% CI) or %	Mean (95% CI) or %	Mean (95% CI) or %
*N*	13 622	2168	2119	3682	5031
Sex, % males	51.8	15.6	44.0	24.6	21.6
Age, y	57.8 (57.6, 57.9)	56.6 (56.2, 56.9)	58.0 (57.7, 58.3)	58.2 (57.9, 58.4)	59.0 (58.8, 59.2)
BMI, kg/m^2^	25.4 (25.3, 25.5)	26.2 (26.0, 26.3)	26.1 (26.0, 26.3)	25.5 (25.4, 25.6)	25.9 (25.8, 26.1)
Waist circumference, cm	83.4 (83.2, 83.5)	84.4 (84.0, 84.8)	85.3 (84.9, 85.8)	83.2 (82.9, 83.5)	83.7 (83.5, 84.0)
Body fat, %	26.6 (26.5, 26.7)	27.2 (27.0, 27.4)	27.2 (27.0, 27.4)	26.7 (26.5, 26.8)	27.1 (26.9, 27.2)
Hb A_1c_, %	4.84 (4.82, 4.85)	4.79 (4.74, 4.83)	4.79 (4.74, 4.84)	4.80 (4.77, 4.84)	4.77 (4.75, 4.80)
High plasma glucose, > 5.6 mmol/L, %	47.1	37.1	49.3	40.6	39.5
HOMA-IR	1.61 (1.56, 1.66)	1.51 (1.40, 1.63)	1.91 (1.78, 2.05)	1.48 (1.39, 1.58)	1.58 (1.50, 1.66)
LTPA, < 7.5 METh/week, %	10.7	6.7	13.3	8.3	6.7
Alcohol, zero consumers, %	5.1	6.9	9.0	6.9	6.6
Smoking, current smoker, %	32.5	21.0	31.5	27.3	19.8
Education, university degree, %	13.0	21.0	11.2	16.9	15.2
Energy misreporters, %	15.5	33.7	17.5	16.7	20.6
Dietary change in the past, %	17.8	42.3	23.3	20.5	26.9
EAT-Lancet index, points	17.0 (16.9, 17.0)	21.0 (20.9, 21.2)	16.5 (16.4, 16.6)	18.2 (18.1, 18.3)	19.4 (19.3, 19.5)
***Dietary variables***					
Energy, kcal/d	2365 (2355, 2374)	1967 (1944, 1991)	2349 (2325, 2373)	2261 (2243, 2279)	2166 (2151, 2182)
Alcohol, E%	3.42 (3.36, 3.48)	3.34 (3.19, 3.49)	2.75 (2.61, 2.90)	3.30 (3.18, 3.41)	3.14 (3.05, 3.24)
Protein, E%	15.8 (15.7, 15.8)	17.1 (17.0, 17.2)	14.6 (14.5, 14.7)	15.5 (15.5, 15.6)	15.8 (15.7, 15.8)
Fat, E%	41.1 (41.0, 41.2)	36.3 (36.1, 36.6)	37.0 (36.8, 37.2)	37.5 (37.3, 37.7)	36.7 (36.5, 36.8)
Carbohydrates, E%	43.2 (43.1, 43.3)	46.6 (46.4, 46.8)	48.4 (48.2, 48.7)	47.0 (46.8, 47.1)	47.6 (47.4, 47.7)
Monosaccharides, E%	5.9 (5.8, 5.9)	9.3 (9.2, 9.4)	8.2 (8.1, 8.3)	9.2 (9.1, 9.3)	9.2 (9.2, 9.3)
Disaccharides, E%	12.4 (12.4, 12.5)	12.0 (11.8, 12.2)	17.0 (16.9, 17.2)	13.2 (13.1, 13.3)	13.0 (12.9, 13.1)
Sucrose, E%	8.0 (7.9, 8.0)	7.8 (7.7, 7.9)	12.9 (12.8, 13.0)	8.9 (8.8, 9.0)	8.8 (8.7, 8.9)
Added sugar, E%	10.0 (9.9, 10.1)	8.0 (7.8, 8.1)	16.0 (15.9, 16.2)	9.5 (9.4, 9.6)	9.3 (9.2, 9.4)
Fiber, g/1000 kcal	8.4 (8.3, 8.4)	12.7 (12.6, 12.8)	8.2 (8.1, 8.3)	9.2 (9.1, 9.2)	11.1 (11.0, 11.1)
Fish, g/1000 kcal	17.8 (17.6, 18.1)	23.9 (23.3, 24.5)	16.3 (15.7, 16.9)	18.4 (18.0, 18.9)	19.6 (19.2, 20.0)
Red meat, g/1000 kcal	54.3 (53.9, 54.6)	47.7 (46.8, 48.6)	51.5 (50.6, 52.4)	49.0 (48.3, 49.7)	48.3 (47.7, 48.9)
Coffee, g/1000 kcal	254 (251, 258)	245 (237, 254)	220 (212, 229)	219 (213, 226)	231 (226, 237)
***Cluster variables (g/1000 kcal)***					
Potato, boiled or baked	48.2 (47.8, 48.7)	41.7 (40.5, 42.8)	44.1 (43.0, 45.3)	43.4 (42.6, 44.3)	44.4 (43.6, 45.2)
Potato, deep-fried + fried	8.41 (8.22, 8.60)	5.52 (5.04, 6.00)	8.07 (7.60, 8.53)	7.24 (6.89, 7.60)	6.58 (6.27, 6.89)
Fruits	61.0 (60.3, 61.8)	142.8 (140.9, 144.6)	77.8 (76.0, 79.6)	88.4 (87.1, 89.8)	170.8 (169.6, 172.0)
Vegetables	68.4 (67.8, 69.0)	192.7 (191.2, 194.2)	71.8 (70.3, 73.3)	81.5 (80.4, 82.7)	87.2 (86.2, 88.2)
Juice	11.2 (10.7, 11.8)	22.2 (20.8, 23.5)	23.0 (21.6, 24.4)	113.8 (112.8, 114.9)	17.7 (16.8, 18.6)
Sugar-sweetened beverages	20.2 (19.5, 20.9)	14.8 (13.1, 16.6)	196.9 (195.2, 198.7)	21.9 (20.6, 23.2)	20.4 (19.2, 21.5)
Pastries	17.8 (17.6, 18.0)	13.5 (13.0, 14.0)	16.3 (15.8, 16.8)	16.2 (15.8, 16.6)	16.3 (16.0, 16.7)
Chocolate	3.80 (3.72, 3.88)	2.64 (2.44, 2.83)	3.23 (3.04, 3.43)	3.19 (3.04, 3.34)	3.14 (3.01, 3.26)
Sweets	3.01 (2.92, 3.10)	1.88 (1.66, 2.11)	3.27 (3.05, 3.49)	2.49 (2.32, 2.65)	2.45 (2.30, 2.59)
Table sugar	4.48 (4.39, 4.58)	2.54 (2.30, 2.78)	4.56 (4.32, 4.80)	3.69 (3.51, 3.87)	3.21 (3.06, 3.37)
Ice cream	4.96 (4.83, 5.09)	5.94 (5.60, 6.28)	5.76 (5.43, 6.09)	5.45 (5.20, 5.71)	6.16 (5.94, 6.38)
Marmalade/honey/jam	7.59 (7.46, 7.72)	6.13 (5.81, 6.45)	7.29 (7.00, 7.61)	7.46 (7.22, 7.70)	7.35 (7.15, 7.56)
Ketchup	0.87 (0.84, 0.89)	0.79 (0.73, 0.85)	0.84 (0.78, 0.90)	0.83 (0.79, 0.88)	0.75 (0.71, 0.79)
Flour	3.41 (3.35, 3.46)	3.58 (3.44, 3.71)	3.16 (3.03, 3.30)	3.55 (3.45, 3.65)	3.63 (3.54, 3.72)
Grains/cereals, < 10% fiber	4.50 (4.39, 4.61)	5.77 (5.50, 6.04)	3.89 (3.62, 4.16)	5.00 (4.79, 5.21)	5.40 (5.22, 5.58)
Grains/cereals, ≥ 10% fiber	0.05 (0.04, 0.06)	0.08 (0.05, 0.11)	0.06 (0.03, 0.08)	0.04 (0.02, 0.06)	0.06 (0.04, 0.08)
Soft bread, < 4.5% fiber	30.5 (30.2, 30.9)	19.3 (18.4, 20.2)	28.1 (27.2, 29.0)	23.7 (23.0, 24.4)	22.3 (21.7, 22.9)
Soft bread, ≥ 4.5% fiber	16.3 (16.0, 16.6)	18.8 (18.0, 19.6)	13.4 (12.6, 14.2)	16.8 (16.2, 17.5)	17.9 (17.3, 18.4)
Crisp bread, < 10% fiber	2.32 (2.26, 2.38)	2.30 (2.15, 2.45)	2.45 (2.30, 2.60)	2.21 (2.10, 2.33)	2.31 (2.21, 2.41)
Crisp bread, ≥ 10% fiber	4.98 (4.87, 5.09)	7.25 (6.97, 7.52)	4.55 (4.28, 4.82)	5.54 (5.33, 5.75)	6.32 (6.14, 6.50)
Rice/pasta	5.09 (4.99, 5.19)	6.93 (6.68, 7.19)	4.96 (4.71, 5.21)	5.38 (5.19, 5.57)	5.55 (5.38, 5.72)

A general linear model was used for continuous variables. Values are expressed in means and confidence intervals, and were adjusted for age and sex. *Age* was adjusted for sex. *EAT-Lancet index* was adjusted for age, sex, and energy intake. *Energy (kcal/d)* was adjusted for age, sex, diet method version, and season. All other diet-related variables were adjusted for age, sex, diet method version, season, and energy intake. Cross tabulation was used for categorical variables, and values are expressed in percentages

^1^*n*=26,587 for BMI, *n* = 26,576 for waist circumference, *n* = 26,468 for body fat, *n* = 5,046 for HbA_1c_, *n* = 5,045 for high plasma glucose, *n* = 4,654 for HOMA-IR, *n* = 26,597 for dietary change in the past, and *n* = 24,631 for EAT-Lancet index

^2^E%, percentage energy intake; HOMA-IR, homoeostatic model assessment of insulin resistance; LTPA, leisure-time physical activity; METh, hours of metabolic equivalent of task

The *high refined carbohydrates/low fruit & vegetables* cluster was characterized by a high intake of pastries and low-fiber soft bread, and a low intake of fruit and vegetables (Table 1). The *high vegetables/low added sugar* cluster was characterized by a high intake of vegetables, and a low intake of sweet foods, including pastries, sugar-sweetened beverages, chocolate, sweets, and table sugar. The *high sugar-sweetened beverages* cluster was characterized by a high intake of sugar-sweetened beverages, while also having a low intake of fiber-rich soft bread and fiber-rich crisp bread. The *high juice* cluster was characterized by a high juice intake and the *high fruit* cluster by a high fruit intake.

For non-clustered variables, the *high refined carbohydrates/low fruit & vegetables* cluster had the highest intake of red meat, the highest proportion of males and current smokers, the lowest BMI, and the lowest proportion of potential energy misreporters and diet changers (Table 1). The *high vegetables/low added sugar* cluster had the highest intake of fiber and fish, the lowest proportion of males and high plasma glucose, the highest EAT-Lancet index score, and the highest proportion of university degrees, potential energy misreporters, and diet changers. The *high sugar-sweetened beverages* cluster had the lowest intake of fiber and fish, the highest waist circumference, the lowest EAT-Lancet index score, and the highest proportion of high plasma glucose and zero-consumers of alcohol. The *high juice* cluster had the lowest waist circumference. The *high fruit* cluster had the highest mean age and the lowest proportion of current smokers.

### Associations between clusters and incidence of type 2 diabetes

In total, there were 4046 participants who developed type 2 diabetes during a mean follow-up of 18.4 years (SD 6.4 years) (Table 2). In the fully adjusted model, there was a significantly lower risk of developing type 2 diabetes in the *high fruit* cluster compared to the *high refined carbohydrates/low fruit & vegetables* cluster (HR 0.86; 95% CI 0.78, 0.94). No other significant associations were identified. In the *high sugar-sweetened beverages* cluster, there was a significant positive association with incident type 2 diabetes in the basic model only (HR 1.13; 95% CI 1.01, 1.27). No interaction with sex was identified for any of the clusters (Supplemental Table 1). Hence, associations were similar for males and for females.

**Table 2:** Hazard ratios (95% CI) of incident type 2 diabetes by clusters of carbohydrate-rich foods in participants in the Malmö Diet and Cancer cohort (*n* = 26 622)

Variable	*n* total/*n* cases/person-years	Model 1^1^	Model 2^2^	Model 3^3^	Model 4^4^
Cluster membership					
High refined carbs/ low fruit & veg	13,622/2193/246,436	1.00	1.00	1.00	1.00
High vegetables/ Low added sugar	2168/334/42,289	1.02 (0.90, 1.15)	1.12 (0.99, 1.26)	0.98 (0.87, 1.11)	0.99 (0.88, 1.12)
High sugar-sweetened beverages	2119/355/36,715	1.13 (1.01, 1.27)*	1.08 (0.96, 1.21)	0.98 (0.87, 1.09)	0.97 (0.86, 1.08)
High juice	3682/498/68,606	0.91 (0.83, 1.01)	0.96 (0.87, 1.06)	0.95 (0.86, 1.05)	0.95 (0.86, 1.05)
High fruit	5031/666/95,138	0.87 (0.80, 0.96)*	0.93 (0.84, 1.01)	0.85 (0.78, 0.93)*	0.86 (0.78, 0.94)*

^1^adjusted for age, sex, diet-method version, season, and total energy intake

^2^adjusted for age, sex, diet-method version, season, total energy intake, physical activity, alcohol habits, smoking, and education

^3^adjusted for age, sex, diet-method version, season, total energy intake, physical activity, alcohol habits, smoking, education, and BMI.

^4^adjusted for age, sex, diet-method version, season, total energy intake, physical activity, alcohol habits, smoking, education, coffee, red meat, and BMI.

^*^P < 0.05

### Sensitivity analyses

Excluding potential energy misreporters (18% of the study sample), diet changers (22%), or participants who developed diabetes within two years (1.3%) did not alter the findings (Supplemental Tables 2, 3 & 4).

## Discussion

In our fully-adjusted model, we identified a significant inverse association between a *high fruit* cluster and incidence of type 2 diabetes in a large cohort in southern Sweden. For the *high sugar-sweetened beverages* cluster, a significant positive association was identified in our basic model only. No associations could be identified for the *high vegetables/low added sugar* and *high juice* clusters.

The current findings provide valuable information in support of an inverse association between fruit intake and type 2 diabetes risk. While previous studies have identified inverse associations by studying fruits independently, or as part of dietary patterns that emphasize a higher fruit intake among other healthy food choices, this is the first study to our knowledge to have identified an inverse association with a dietary pattern that was primarily defined by a high fruit intake [2, 4].

The lower risk for the *high fruit* cluster confirmed previous findings in our cohort, where we identified a significant inverse association between fruit intake and incidence of type 2 diabetes [9]. We have previously also identified an association between a higher intake of monosaccharides, which was strongly associated with fruit intake, and lower risk of type 2 diabetes. In our current study, while the *high fruit* cluster did consume a higher energy percentage of monosaccharides compared to the reference cluster, the intake was not significantly higher than for all other clusters. In the previous study we also identified strong correlations between fruit intake and intake of other carbohydrate-rich foods, highlighting the need to conduct this study of associations between clusters of carbohydrate-rich foods and type 2 diabetes risk.

Both fruit and vegetable intake have been independently associated with type 2 diabetes risk in several previous studies. Zheng et al. [35] identified an inverse association with type 2 diabetes risk with higher fruit and vegetable intake already from levels below recommended daily intake, while a meta-analysis by Schwingschackl et al. [2] identified a dose-response inverse association with incident type 2 diabetes for daily fruit intake of up to 200–300 g, and for daily vegetable intake up to 300 g. In a previous study we concluded that a median daily fruit intake of 357 g per day in the highest quintile was associated with lower incidence of type 2 diabetes in the MDCS cohort, as well as, in males only, for a median daily vegetable intake of 301 g per day in the highest quintile [9]. However, no association was found for vegetable intake in females, and in our current study we did not find any associations for the *high vegetables/low added sugar* cluster with type 2 diabetes incidence.

Fruits and vegetables have low energy density and a high content of micronutrients, fiber and polyphenols, all of which may contribute to their potential protective effect. A meta-analysis by Rienks et al. [36] found an association with type 2 diabetes risk for intake of polyphenols, and for flavonoids in particular. However, more studies are needed to determine the potential role for polyphenols in the prevention of type 2 diabetes.

In comparison to the reference cluster, the *high fruit* cluster also had a higher intake of vegetables, juice, ice cream, flour, and high fiber soft bread and crisp bread, and a lower intake of potato, pastries, chocolate, sweets, table sugar, marmalade/honey/jam, ketchup, and low fiber soft bread and crisp bread. Thus, these foods may also have contributed to the significant difference between the clusters, and several of them have previously been associated with incident type 2 diabetes in the MDCS cohort [9].

Associations between dietary patterns and incidence type 2 diabetes have been studied previously in our cohort. Ericson et al. [7] applied principal component analysis to identify dietary patterns in the total diet of the MDCS cohort and found a significant inverse association between a health-conscious pattern and type 2 diabetes risk. One of the characteristics of this dietary pattern was a high fruit intake. In other studies, plant-based dietary patterns have been found to be associated with a reduced risk of type 2 diabetes, particularly when intake of healthy foods such as fruit and vegetables was emphasized [3]. This may be due a higher intake of nutrient-dense foods with low energy density while limiting or excluding the intake of red and processed meat. Similarly, diets with an emphasis on higher intake of fruit and vegetables, including the Mediterranean diet, the DASH diet, and the Alternative Healthy Eating Index, have all been inversely associated with type 2 diabetes risk [4]. Studies have also shown that the dietary glycemic index in the diet may play a role in the prevention of type 2 diabetes [6, 37, 38]. Many dietary patterns associated with a lower incidence of type 2 diabetes emphasize the intake of foods that are often low in glycemic index, including wholegrains, fruits and vegetables.

Few studies have applied cluster analysis to identify dietary patterns and studied the associations with incident type 2 diabetes in prospective cohorts, and they have shown inconsistent results. Villegas et al. [12] applied K-means cluster analysis to study associations with type 2 diabetes in middle-aged Chinese females. They found an inverse association for a dietary pattern defined by a high intake of dairy milk, and a low intake of staple foods, such as rice, and soy foods, compared to the reference cluster. Brunner et al. [13] used cluster analysis to examine dietary patterns of middle-aged British adults. In comparison with an *unhealthy* dietary pattern, a *healthy* pattern defined by a higher intake of fruits and nuts, vegetables, high-fiber bread, and low-fat dairy, and a lower intake of red meat and alcohol was associated with a lower risk of diabetes. A study by Hsiao et al. [14] explored dietary patterns in a small population of older American adults (75 years and older) using cluster analysis. The authors did not identify an increased risk of type 2 diabetes for a *sweets & dairy* and a *Western* dietary pattern, compared to a *health-conscious* dietary pattern.

In our study, we could not identify any significant associations for neither the *high juice* nor the *high sugar-sweetened beverages* cluster in our final model. The findings are similar to a previous study in our cohort where no associations for a higher intake of sugar-sweetened beverages or a higher juice intake with type 2 diabetes incidence could be identified [9]. Meanwhile, several other studies have identified significant positive associations for sugar-sweetened beverages and type 2 diabetes risk, while findings have been less clear for juice intake [2, 39, 40].

K-means cluster analysis is one of the most commonly applied clustering algorithms for identifying dietary patterns and has been found to be superior to other common methods of clustering [15, 41, 42]. However, there is no gold standard approach to decide the exact number of clusters to include in the analysis. Our decision was based on the size and distribution of participants in each cluster, and the distinguishable dietary patterns that emerged. There is also a subjective decision in determining how many and what variables to include in the analysis, and whether certain variables should be merged into a larger variable, such as foods into a larger food group. For this study we were specifically interested in studying clusters of carbohydrate-rich foods and their association with incidence of type 2 diabetes. Thus, we did not include other food variables potentially associated with type 2 diabetes risk in our cluster analysis. Different approaches can also be seen as for whether to energy-adjust and standardise the dietary variables. We decided to energy-adjust but not to standardize, by using e.g. Z-scores, as this may result in poor dietary patterns when foods eaten in smaller quantities are given disproportionate weights to foods eaten in larger quantities. Lastly, a decision also has to be made on what cluster to assign as the reference cluster in the analysis, and to interpret the findings accordingly. We applied the *high refined carbohydrates/low fruit & vegetables* cluster as the reference, as it encompassed more than half of the participants and was not primarily characterized by the intake of one specific food variable.

A major strength in our study is the large study population combined with the long follow-up time, resulting in a large number of type 2 diabetes cases. The large population size enabled the identification of five distinct dietary patterns of carbohydrate-rich foods, with a sizable number of individuals in each cluster, while the large number of cases increased the ability to identify associations between the derived clusters and type 2 diabetes risk. The prospective design of this study limited the risk of reverse causation and reduced selection bias. Another strength is the relatively high validity of the three-part diet method applied in this study. The comprehensive data on covariates provided the opportunity to make ample adjustment for possible confounders in our models. However, residual confounding due to unknown factors may still be present. This study also relied solely on self-reported dietary data that was only collected at baseline. Although past diet changers were excluded in one of the sensitivity analyses, we cannot rule out that there may be participants who have made major changes to their diets since baseline examinations. Additionally, relying on self-reported dietary data provides a well-known risk of measurement error due to misreporting, leading to a misclassification of exposure. Hence, we performed an additional sensitivity analysis where potential misreporters of energy intake were excluded, which did not alter our findings. Generalizability of the findings may be limited due to a less diverse population and different eating habits compared to present-day Malmo, and some limitations in representativity [16]. Meanwhile, the study population constituted a large, socio-demographically representative sample, and could be divided into five distinct and substantial clusters. Lastly, despite excluding participants with type 1 diabetes, latent autoimmune diabetes in adults, secondary diabetes or other diabetes condition from the study, about half of all included cases were registered as being of unknown type. However, considering the age of the participants and the exclusion of baseline diabetes cases it is likely to assume that the vast majority of these cases were of type 2. Removing these cases would have greatly reduced the power of the study.

In conclusion, we identified a reduced risk of incident type 2 diabetes with a dietary pattern primarily defined by a high fruit intake. To our knowledge, this is the first study that has investigated the associations between clusters of carbohydrate-rich foods and incidence of type 2 diabetes. The findings provide additional evidence that a dietary pattern high in fruits may lower the risk of developing type 2 diabetes. Future studies are needed to explore this association further.

### Electronic supplementary material

Below is the link to the electronic supplementary material.

Supplementary Material 1

## Data Availability

The data are available after application to the MDC Steering Committee (http://malmo-kohorter.lu.se/malmo-cohorts).

## References

[1] Federation ID (2021). IDF Diabetes Atlas.

[2] Schwingshackl L, Hoffmann G, Lampousi AM, Knuppel S, Iqbal K, Schwedhelm C, Bechthold A, Schlesinger S, Boeing H (2017). Food groups and risk of type 2 Diabetes Mellitus: a systematic review and meta-analysis of prospective studies. Eur J Epidemiol.

[3] Qian F, Liu G, Hu FB, Bhupathiraju SN, Sun Q (2019). Association between Plant-based dietary patterns and risk of type 2 Diabetes: a systematic review and Meta-analysis. JAMA Intern Med.

[4] Jannasch F, Kroger J, Schulze MB (2017). Dietary patterns and type 2 Diabetes: a systematic literature review and Meta-analysis of prospective studies. J Nutr.

[5] Uusitupa M, Khan TA, Viguiliouk E, Kahleova H, Rivellese AA, Hermansen K, Pfeiffer A, Thanopoulou A, Salas-Salvado J, Schwab U et al. (2019) Prevention of type 2 Diabetes by Lifestyle changes: a systematic review and Meta-analysis. Nutrients 11. 10.3390/nu11112611. 10.3390/nu11112611 PMC6893436 31683759

[6] Ley SH, Hamdy O, Mohan V, Hu FB (2014). Prevention and management of type 2 Diabetes: dietary components and nutritional strategies. Lancet.

[7] Ericson U, Brunkwall L, Alves Dias J, Drake I, Hellstrand S, Gullberg B, Sonestedt E, Nilsson PM, Wirfalt E, Orho-Melander M (2019). Food patterns in relation to weight change and incidence of type 2 Diabetes, coronary events and Stroke in the Malmo Diet and Cancer cohort. Eur J Nutr.

[8] Mandalazi E, Drake I, Wirfalt E, Orho-Melander M, Sonestedt E. A high Diet Quality based on dietary recommendations is not Associated with Lower incidence of type 2 Diabetes in the Malmo Diet and Cancer Cohort. Int J Mol Sci. 2016;17. 10.3390/ijms17060901. 10.3390/ijms17060901 PMC4926435 27338354

[9] Olsson K, Ramne S, Gonzalez-Padilla E, Ericson U, Sonestedt E. Associations of carbohydrates and carbohydrate-rich foods with incidence of type 2 Diabetes. Br J Nutr. 2020;1–11. 10.1017/S0007114520005140. 10.1017/S0007114520005140 33355062

[10] Liu J, Rehm CD, Shi P, McKeown NM, Mozaffarian D, Micha R (2020). A comparison of different practical indices for assessing carbohydrate quality among carbohydrate-rich processed products in the US. PLoS ONE.

[11] Hu FB (2002). Dietary pattern analysis: a new direction in nutritional epidemiology. Curr Opin Lipidol.

[12] Villegas R, Yang G, Gao YT, Cai H, Li H, Zheng W, Shu XO (2010). Dietary patterns are associated with lower incidence of type 2 Diabetes in middle-aged women: the Shanghai women’s Health Study. Int J Epidemiol.

[13] Brunner EJ, Mosdol A, Witte DR, Martikainen P, Stafford M, Shipley MJ, Marmot MG (2008). Dietary patterns and 15-y risks of major coronary events, Diabetes, and mortality. Am J Clin Nutr.

[14] Hsiao PY, Mitchell DC, Coffman DL, Craig Wood G, Hartman TJ, Still C, Jensen GL (2013). Dietary patterns and relationship to obesity-related health outcomes and mortality in adults 75 years of age or greater. J Nutr Health Aging.

[15] Devlin UM, McNulty BA, Nugent AP, Gibney MJ (2012). The use of cluster analysis to derive dietary patterns: methodological considerations, reproducibility, validity and the effect of energy mis-reporting. Proc Nutr Soc.

[16] Manjer J, Carlsson S, Elmstahl S, Gullberg B, Janzon L, Lindstrom M, Mattisson I, Berglund G (2001). The Malmo Diet and Cancer Study: representativity, cancer incidence and mortality in participants and non-participants. Eur J Cancer Prev.

[17] Riboli E, Elmstahl S, Saracci R, Gullberg B, Lindgarde F (1997). The Malmo Food Study: validity of two dietary assessment methods for measuring nutrient intake. Int J Epidemiol.

[18] Elmstahl S, Riboli E, Lindgarde F, Gullberg B, Saracci R (1996). The Malmo Food Study: the relative validity of a modified diet history method and an extensive food frequency questionnaire for measuring food intake. Eur J Clin Nutr.

[19] Berglund G, Elmstahl S, Janzon L, Larsson SA (1993). The Malmo Diet and Cancer Study. Design and feasibility. J Intern Med.

[20] Wirfalt E, Mattisson I, Johansson U, Gullberg B, Wallstrom P, Berglund G (2002). A methodological report from the Malmo Diet and Cancer study: development and evaluation of altered routines in dietary data processing. Nutr J.

[21] Ramne S, Alves Dias J, Gonzalez-Padilla E, Olsson K, Lindahl B, Engstrom G, Ericson U, Johansson I, Sonestedt E (2019). Association between added sugar intake and mortality is nonlinear and dependent on sugar source in 2 Swedish population-based prospective cohorts. Am J Clin Nutr.

[22] Stubbendorff A, Sonestedt E, Ramne S, Drake I, Hallstrom E, Ericson U (2022). Development of an EAT-Lancet index and its relation to mortality in a Swedish population. Am J Clin Nutr.

[23] Willett W, Rockstrom J, Loken B, Springmann M, Lang T, Vermeulen S, Garnett T, Tilman D, DeClerck F, Wood A (2019). Food in the Anthropocene: the EAT-Lancet Commission on healthy diets from sustainable food systems. Lancet.

[24] Mutie PM, Drake I, Ericson U, Teleka S, Schulz CA, Stocks T, Sonestedt E (2020). Different domains of self-reported physical activity and risk of type 2 Diabetes in a population-based Swedish cohort: the Malmo diet and Cancer study. BMC Public Health.

[25] Mattisson I, Wirfalt E, Aronsson CA, Wallstrom P, Sonestedt E, Gullberg B, Berglund G (2005). Misreporting of energy: prevalence, characteristics of misreporters and influence on observed risk estimates in the Malmo Diet and Cancer cohort. Br J Nutr.

[26] Sonestedt E, Wirfalt E, Gullberg B, Berglund G (2005). Past food habit change is related to obesity, lifestyle and socio-economic factors in the Malmo Diet and Cancer Cohort. Public Health Nutr.

[27] Hedblad B, Nilsson P, Janzon L, Berglund G (2000). Relation between insulin resistance and carotid intima-media thickness and stenosis in non-diabetic subjects. Results from a cross-sectional study in Malmo, Sweden. Diabet Med.

[28] SND. Scania Diabetes Registry Gothenburg. : University of Gothenburg; Swedish National Data Service. https://snd.gu.se/en/catalogue/study/ext0074. Accessed 16 June 2022.

[29] NDR. The Swedish National Diabetes Register Gothenburg. Registercentrum Västra Götaland. https://www.ndr.nu/#/english. Accessed 16 June 2022.

[30] Socialstyrelsen. Statistical Databases Stockholm: National Board of Health and Welfare. https://www.socialstyrelsen.se/en/statistics-and-data/statistics/statistical-databases/. Accessed 16 June 2022.

[31] Socialstyrelsen, The National Patient Register Stockholm. National Board of Health and Welfare. https://www.socialstyrelsen.se/en/statistics-and-data/registers/register-information/the-national-patient-register/. Accessed 16 June 2022.

[32] Socialstyrelsen. Läkemedelsregistret Stockholm: National Board of Health and Welfare. https://www.socialstyrelsen.se/statistik-och-data/register/alla-register/lakemedelsregistret/. Accessed 16 June 2022.

[33] Carlstrom M, Larsson SC (2018). Coffee consumption and reduced risk of developing type 2 Diabetes: a systematic review with meta-analysis. Nutr Rev.

[34] Baron RM, Kenny DA (1986). The moderator-mediator variable distinction in social psychological research: conceptual, strategic, and statistical considerations. J Pers Soc Psychol.

[35] Zheng JS, Sharp SJ, Imamura F, Chowdhury R, Gundersen TE, Steur M, Sluijs I, van der Schouw YT, Agudo A, Aune D (2020). Association of plasma biomarkers of fruit and vegetable intake with incident type 2 Diabetes: EPIC-InterAct case-cohort study in eight European countries. BMJ.

[36] Rienks J, Barbaresko J, Oluwagbemigun K, Schmid M, Nothlings U (2018). Polyphenol exposure and risk of type 2 Diabetes: dose-response meta-analyses and systematic review of prospective cohort studies. Am J Clin Nutr.

[37] Livesey G, Taylor R, Livesey HF, Buyken AE, Jenkins DJA, Augustin LSA, Sievenpiper JL, Barclay AW, Liu S, Wolever TMS et al. (2019) Dietary glycemic index and load and the risk of type 2 Diabetes: a systematic review and updated Meta-analyses of prospective cohort studies. Nutrients 11. 10.3390/nu11061280. 10.3390/nu11061280 PMC6627334 31195724

[38] Livesey G, Taylor R, Livesey HF, Buyken AE, Jenkins DJA, Augustin LSA, Sievenpiper JL, Barclay AW, Liu S, Wolever TMS et al. (2019) Dietary glycemic index and load and the risk of type 2 Diabetes: Assessment of Causal relations. Nutrients 11. 10.3390/nu11061436. 10.3390/nu11061436 PMC6628270 31242690

[39] Imamura F, O’Connor L, Ye Z, Mursu J, Hayashino Y, Bhupathiraju SN, Forouhi NG (2015). Consumption of sugar sweetened beverages, artificially sweetened beverages, and fruit juice and incidence of type 2 Diabetes: systematic review, meta-analysis, and estimation of population attributable fraction. BMJ.

[40] Ma L, Hu Y, Alperet DJ, Liu G, Malik V, Manson JE, Rimm EB, Hu FB, Sun Q (2023). Beverage consumption and mortality among adults with type 2 Diabetes: prospective cohort study. BMJ.

[41] Fransen HP, May AM, Stricker MD, Boer JM, Hennig C, Rosseel Y, Ocke MC, Peeters PH, Beulens JW (2014). A posteriori dietary patterns: how many patterns to retain?. J Nutr.

[42] Sauvageot N, Schritz A, Leite S, Alkerwi A, Stranges S, Zannad F, Streel S, Hoge A, Donneau AF, Albert A (2017). Stability-based validation of dietary patterns obtained by cluster analysis. Nutr J.

