# Supplementary material for: Clusters of carbohydrate-rich foods and associations with type 2 diabetes incidence: a prospective cohort study
Source: Nutr J. 2023 Dec 18;22:71. doi: 10.1186/s12937-023-00906-0 (PMC10726530; doi:10.1186/s12937-023-00906-0)
Supplement: Supplementary file 1 — Supplementary Material 1 [file 12937_2023_906_MOESM1_ESM.docx]

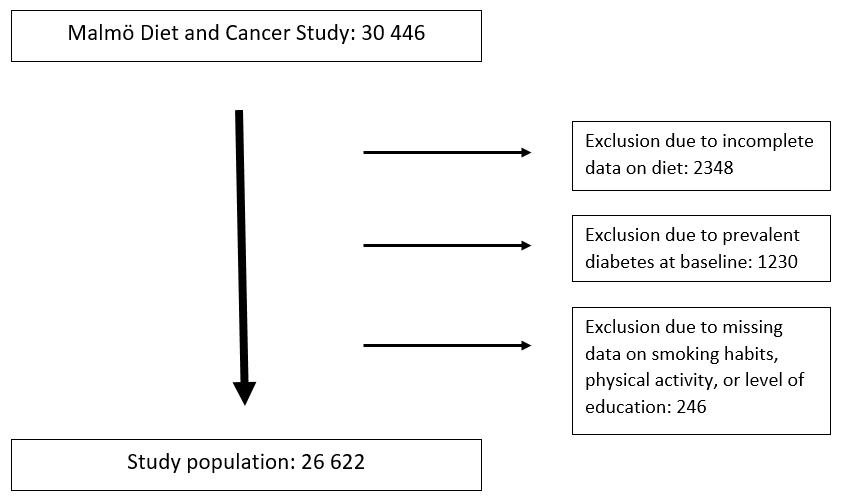


**Supplemental Figure 1.** Flowchart illustrating the selection process of participants in the study population.


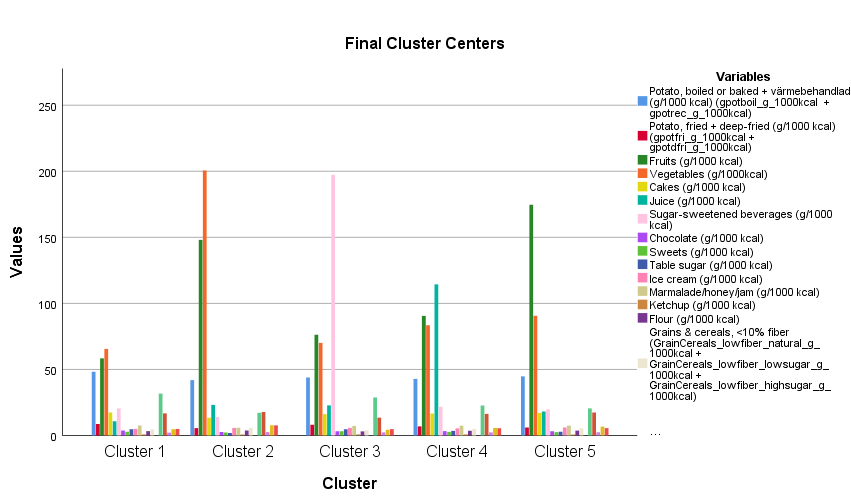
**Supplemental Figure 2.** Clusters identified through K-means cluster analysis of carbohydrate-rich foods in the Malmö Diet and Cancer cohort (n=26 622).

**Supplemental Table 1.** *P* for interaction with sex for each cluster compared to the reference cluster in participants in the Malmö Diet and Cancer cohort.

| Variable | Model 4^a^ |
| --- | --- |
| Cluster membership |  |
| High vegetables/Low added sugar | 0.94 |
| High sugar-sweetened beverages | 0.56 |
| High juice | 0.75 |
| High fruit | 0.45 |

^a^adjusted for sex, age, diet-method version, season, total energy intake, physical activity, alcohol habits, smoking, education, coffee, red meat, and BMI.

**Supplemental Table 2.** Sensitivity analysis of hazard ratios (95% CI) of incident type 2 diabetes by clusters of carbohydrate-rich foods in participants in the Malmö Diet and Cancer cohort, excluding participants who had under- or overreported their energy intake (compared to their level of physical activity) (*n* = 21,758).

| Variable | Model 4  (excluding misreporters)^a^ |
| --- | --- |
| Cluster membership |  |
| High refined carbs/Low fruit & veg | 1.00 |
| High vegetables/Low added sugar | 1.03 (0.89, 1.20) |
| High sugar-sweetened beverages | 0.94 (0.83, 1.07) |
| High juice | 0.99 (0.89, 1.11) |
| High fruit | 0.86 (0.77, 0.95)* |

^a^adjusted for sex, age, diet-method version, season, total energy intake, physical activity, alcohol habits, smoking, education, coffee, red meat, and body mass index (BMI).

^*^*P*<0.05

**Supplemental Table 3.** Sensitivity analysis of hazard ratios (95% CI) of incident type 2 diabetes by clusters of carbohydrate-rich foods in participants in the Malmö Diet and Cancer cohort, excluding participants who had made recent changes to their diet (due to e.g. illness) (*n* = 20,660).

| Variable | Model 4  (excluding diet changers)^a^ |
| --- | --- |
| Cluster membership |  |
| High refined carbs/Low fruit & veg | 1.00 |
| High vegetables/Low added sugar | 0.90 (0.77, 1.07) |
| High sugar-sweetened beverages | 0.92 (0.80, 1.05) |
| High juice | 0.92 (0.82, 1.03) |
| High fruit | 0.84 (0.75, 0.93)* |

^a^adjusted for sex, age, diet-method version, season, total energy intake, physical activity, alcohol habits, smoking, education, coffee, red meat, and BMI.

^*^*P*<0.05

**Supplemental Table 4.** Sensitivity analysis of hazard ratios (95% CI) of incident type 2 diabetes by clusters of carbohydrate-rich foods in participants in the Malmö Diet and Cancer cohort, excluding participants with registered diabetes within two years of enrolment in study (*n* = 26,270).

| Variable | Model 4  (excluding diet changers)^a^ |
| --- | --- |
| Cluster membership |  |
| High refined carbs/Low fruit & veg | 1.00 |
| High vegetables/Low added sugar | 0.99 (0.87, 1.12) |
| High sugar-sweetened beverages | 0.92 (0.82, 1.04) |
| High juice | 0.91 (0.82, 1.01) |
| High fruit | 0.85 (0.77, 0.93)* |

^a^adjusted for sex, age, diet-method version, season, total energy intake, physical activity, alcohol habits, smoking, education, coffee, red meat, and BMI.

^*^*P*<0.05
